# Supplementary material for: Correlates of perceived access and implications for health system strengthening – lessons from HIV/AIDS treatment and care services in Ethiopia
Source: PLoS One. 2016 Aug 22;11(8):e0161553. doi: 10.1371/journal.pone.0161553 (PMC4993581; doi:10.1371/journal.pone.0161553)
Supplement: S1 File — (DOCX) [file pone.0161553.s001.docx]

**S1 File Questionnaire**

**A questionnaire for People Living with HIV and Using HIV/AIDS Treatment and Care Services**

**Identification of the Questionnaire (QID)**

This section is must be completed by the interviewer before proceeding to the next sections.

| Q# | Question | Response | Remark/Skip |
| --- | --- | --- | --- |
| 101 | Date of Interview | ______________________ |  |
| 102 | Name of Health Facility | _______________________ |  |
| 103 | Type of Health Facility | Hospital. 1  Health Center. 2 |  |
| 104 | Name of Administrative Zone | Wolaita. 1 |  |
| 105 | Name of administrative woreda/district | _____________________ |  |
| 106 | Name of administrative kebele/village | _____________________ |  |
| 107 | Interviewer’s code | ______________ |  |

**Socio-demographic characteristics of the respondent/participant**

***Instructions***

**To the interviewer**: Read the questions to the participant and circle or write accordingly on the response column. Follow directions provided on the skip column wherever applies.

**To the participant**: I want to ask you general questions about yourself. Please tell me what applies to you.

| **Q#** | **Question** | **Response** | **Remark/Skip** |
| --- | --- | --- | --- |
| 201 | How old are you?  (in years) | ________________ |  |
| 202 | Record sex as observed  (Don’t ask the participant) | Male. 1  Female. 2 |  |
| 203 | Which zone do you live in? | Woliata. 1  Other. 2 | Quit |
| 204 | District [Woreda] or Town administration | ________________ |  |
| 205 | Village/Kebele | ________________ |  |
| 206 | What is your current marital Status? | Never Married. 1  Currently Married. 2  Divorced. 3  Widowed. 4  Separated. 5  Cohabiting.6  Don’t want to mention. 99 |  |
| 207 | What is the highest education you have achieved? | None at all. 1  Basic education (read & write). 2  Primary 1^st^ cycle (grade 1-4). 3  Primary 2^nd^ cycle (grade 5-8). 4  Secondary (grade 9-12). 5  Vocational school. 6  Tertiary (College/University). 7  I don’t want to mention. 99 |  |
| 208 | In total, how many years have you spent in school | _____________ |  |
| 209 | Please tell me your religious affiliation  (Do not read alternatives) | Orthodox Christian. 1  Catholic Christian. 2  Protestant Christian. 3  Muslim. 4  Traditional. 5  Not affiliated to any. 6  Other (specify). 98  ________________  Don’t want to mention. 99 |  |
| 210 | What is your employment status?  (Do not read options) | Paid Work. 1  Self - Employed. 2  Non-paid work. 3  Student. 4  Keeping house/maid. 5  Retired. 6  Unemployed (health reason). 7  Unemployed (other reasons). 8  Other (specify). 9  ___________________  Donations/NGO support. 10 |  |
| 211 | What is your average monthly income in Birr?  (for irregular income, calculate an average) | _________________ |  |
| 212 | How would you categorize your family in terms of income? | Low class. 1  Middle class. 2  High class. 3 |  |
| 213 | What is your family size including you? | ______________ |  |

**General Health and HIV/AIDS**

The following questions ask you about your perceptions related about your health and HIV/AIDS status. Please make your own assessment of the questions and tell me what applies to you.

| Q# | Question | Response | Remark/Skip |
| --- | --- | --- | --- |
| 301 | How would you rate your health?  (Read options. Rate ranges from very poor to very good) | Very Poor. 1  Poor. 2  Neither poor nor good. 3  Good. 4  Very Good. 5 |  |
| 302 | Do you consider yourself currently ill? | Yes. 1  No. 2  I don’t know. 9  I don’t want to mention. 99 |  |
| 303 | In what year did you first test positive for HIV? | ______________ |  |
| 306 | What do you think how you acquired HIV? (How did you get HIV? | Sexual. 1  Injecting drugs. 2  Blood products/transfusion. 3  Sharing sharp materials. 4  I don’t know. 9  Other (specify). 98  ____________________  I don’t want to mention. 99 |  |
| 307 | Have you disclosed your HIV status to anyone? | Yes. 1  No. 2 | Proceed to 309  Skip to 310 |
| 309 | If Yes to #307, to whom have you disclosed? | Partner/spouse. 1  Family members. 2  Friends. 3  Community. 4  Other. 98  ------------------------------- | Skip to 311 |
| 311 | When have you started HIV care (ART/pre-ART)? (month and year) | ----------------------Month  ------------------------Year |  |
| 312 | Are you currently obtaining TB treatment? | Yes. 1  No. 2 |  |

**Patient Health Questionnaire (PHQ9)**

The purpose of the following question is to know how often the issues listed in the table below have bothered you. Please estimate the number of days as follows: Not at all (0 days), Several days (1-6 days), more than half the days (7-11 days) and Nearly every day (12-14 days). Please circle only one number per item.

| 313 | Over the last two weeks, how often have you been bothered by any of the following problems | Not at all | Several days | More than half the days | Nearly every day |
| --- | --- | --- | --- | --- | --- |
| a | Little interest or pleasure in doing things | 0 | 1 | 2 | 3 |
| b | Feeling down, depressed, or hopeless | 0 | 1 | 2 | 3 |
| c | Trouble falling asleep, or sleeping too much | 0 | 1 | 2 | 3 |
| d | Feeling tired or having little energy | 0 | 1 | 2 | 3 |
| e | Poor appetite or overeating | 0 | 1 | 2 | 3 |
| f | Feeling bad about yourself – or that you are a failure or have let yourself or your family down | 0 | 1 | 2 | 3 |
| g | Trouble concentrating on things, such as reading the newspaper or watching television | 0 | 1 | 2 | 3 |
| h | Moving or speaking so slowly that other people could have noticed? Or the opposite – being do fidgety or restless that you have been moving around a lot more than usual | 0 | 1 | 2 | 3 |
| i | Thoughts that you would be better off dead or hurting yourself in some way | 0 | 1 | 2 | 3 |
| 314 | If you scored 1 or above for any problems listed above, how difficult have these problems made it for you to do your work, take care of things at home, or get along with other people? | | | Not difficult at all – 1  Somewhat difficult- 2  Very difficult – 3  Extremely Difficult - 4 | |
| 315 | If your score is 1 or above for any of the above problems, how difficult have these problems made it for you to visit clinics, your appointments with care provider or your choices of health facilities? | | | Not difficult at all – 1  Somewhat difficult- 2  Very difficult – 3  Extremely Difficult - 4 | |

**Health Care Access**

In the following section, I want to ask you about access to health care and your experiences. Please tell me based on your experiences and what you feel about it. Please be aware that there is no right or wrong answer.

| Q# | Question | Response | Remark/Skip |
| --- | --- | --- | --- |
| 501 | How far is this health facility from your residence? (estimate in km) | ________________ |  |
| 502 | What transportation means did you use to reach here? | Walked. 1  Public transport (vehicle). 2  Own vehicle. 3  Animal transport. 4  Walk + Animal transport. 5  Walk + Public transport. 6  Walk + Animal transport + Vehicle. 7  Other (Specify). 98  ______________________ |  |
| 503 | How long did it take you to reach here? ( estimate in minutes) | __________________ |  |
| 504 | How do you rate the difficulty of the landscape on the way to this health facility? | Very difficult. 1  Fairly difficult. 2  Neutral/fair. 3  Fairly easy. 4  Very easy. 5 |  |
| 505 | The following four questions are about how transportation impacts your access to health services. Please tell me how you feel about each of the statements. These questions are adapted from WHOQOL Scale |  |  |
| a | To what extent do you have adequate means of transportation to health care? | Not at all. 1  A little. 2  Moderately. 3  Mostly. 4  Completely. 5 |  |
| b | To what extent do you have problems with transportation to health care? | Not at all. 1  A little. 2  A moderate amount. 3  Very Much. 4  An extreme amount. 5 |  |
| c | How much difficulties with transport restrict your access to health care? | Not at all. 1  A little. 2  A moderate amount. 3  Very Much. 4  An extreme amount. 5 |  |
| d | How satisfied are you with your transport to healthcare? | Very dissatisfied. 1  Dissatisfied. 2  Neutral. 3  Satisfied. 4  Very satisfied. 5 |  |
| 506 | What are other expenses related that you cover while travelling for HIV/AIDS treatment and care services?  (Do not read options. Circle all mentioned) | Transportation. 1  Accommodation. 2  Food. 3  I don’t pay. 4  Others (specify). 98  __________________ |  |
| 508 | Have you ever experienced stigma/discrimination because you are infected with HIV? | Yes. 1  No.2 |  |
| 509 | If Yes to #517, to what extent has stigma/discrimination affected your access to HIV/AIDS treatment and care services? | Not at all. 1  To some extent. 2  Moderately. 3  Severely. 4  Extremely/totally. 5 |  |
| 510 | How do you generally rate access to HIV/AIDS treatment and care services for you?  **(Please rate each of the following questions in relation to your experiences and perceptions about distance from health facility, travel time and waiting time, how you are treated in the health facility and how you see the quality of care in the HIV care unit**.) | Very poor. 1  Fairly poor. 2  Neutral. 3  Fairly good. 4  Very good. 5 |  |

**Part VI. Acceptability and Responsiveness of the Health Care**

In the following questions, I will ask you how acceptable the health services are to you and how you feel about the environment you are treated. Please feel free and tell what you feel about each questions. Please answer with in the response range of **very poor** to **very good**. Very good when it is the best and very poor when it is the worst.

| *601* | How would you rate the responsiveness (the environment in which you are treated) of this healthcare facility in terms of amenities to care? | *Please circle the number that represents your valuation out of five scale as indicated*(circle that applies) | | | | |
| --- | --- | --- | --- | --- | --- | --- |
| *a* | Cleanliness of health care units | *Very poor*  *1* | *Poor*  *2* | *Neutral*  *3* | *Good*  *4* | *Very Good*  *5* |
| *b* | Maintenance of buildings in health care units | *Very poor*  *1* | *Poor*  *2* | *Neutral*  *3* | *Good*  *4* | *Very Good*  *5* |
| *c* | Adequacy of furniture in health care units | *Very poor*  *1* | *Poor*  *2* | *Neutral*  *3* | *Good*  *4* | *Very Good*  *5* |
| *d* | Convenience and attractiveness of the premises of the health care facility | *Very poor*  *1* | *Poor*  *2* | *Neutral*  *3* | *Good*  *4* | *Very Good*  *5* |
| *e* | Access to clean water at health care units | *Very poor*  *1* | *Poor*  *2* | *Neutral*  *3* | *Good*  *4* | *Very Good*  *5* |
| *f* | Cleanliness of toilets in health care units | *Very poor*  *1* | *Poor*  *2* | *Neutral*  *3* | *Good*  *4* | *Very Good*  *5* |
| *g* | Cleanliness of linen in health care units? | *Very poor*  *1* | *Poor*  *2* | *Neutral*  *3* | *Good*  *4* | *Very Good*  *5* |
| *h* | Facilities for people with disabilities in the health care units | *Very poor*  *1* | *Poor*  *2* | *Neutral*  *3* | *Good*  *4* | *Very Good*  *5* |
| *i* | How would you generally rate the health care facility regarding the quality of amenities to care? | *Very poor*  *1* | *Poor*  *2* | *Neutral*  *3* | *Good*  *4* | *Very Good*  *5* |
| *j* | To what extent do your perceptions about basic amenities contribute to your assessment of overall quality of health care services? | *Very poor*  *1* | *Poor*  *2* | *Neutral*  *3* | *Good*  *4* | *Very Good*  *5* |
| *k* | The smell in health care units | *Very bad*  *1* | *Bad*  *2* | *Neutral*  *3* | *Good*  *4* | *Very Good*  *5* |
| *l* | How would you rate the overall convenience of the environment you are treated including cleanliness? | *Very poor*  *1* | *Poor*  *2* | *Neutral*  *3* | *Good*  *4* | *Very Good*  *5* |
| *Proceed to Q602* | | | | | | |

In the following questions, I will ask you how acceptable the health services are to you and how you feel about the way you are treated. Please feel free and tell what you feel about each questions. Please rate how you feel about each of the questions as Never, Sometimes, Usually or Always.

| ***602*** | How do you rate respect for you were granted and orientation you received when you are treated in this health facility? | *Please rate out of four for the following statements*(circle that applies) | | | |  |
| --- | --- | --- | --- | --- | --- | --- |
| ***a*** | How often are you treated with respect in the health care units? | *Never*  *1* | *Sometimes*  *2* | *Usually*  *3* | *Always*  *4* |  |
| ***b*** | How often are you encouraged to discuss your concerns freely? | *Never*  *1* | *Sometimes*  *2* | *Usually*  *3* | *Always*  *4* |  |
| ***c*** | How often are you encouraged to ask questions about diseases, treatment and care? | *Never*  *1* | *Sometimes*  *2* | *Usually*  *3* | *Always*  *4* |  |
| ***d*** | How often is respect shown for the patient’s desire for privacy during treatment and examinations? | *Never*  *1* | *Sometimes*  *2* | *Usually*  *3* | *Always*  *4* |  |
| ***e*** | How often are you provided with information on alternative treatment options? | *Never*  *1* | *Sometimes*  *2* | *Usually*  *3* | *Always*  *4* |  |
| ***f*** | How often are you consulted about your preferences over alternative treatment options? | *Never*  *1* | *Sometimes*  *2* | *Usually*  *3* | *Always*  *4* |  |
| ***g*** | How often is your consent sought before testing or starting treatment? | *Never*  *1* | *Sometimes*  *2* | *Usually*  *3* | *Always*  *4* |  |
| *Proceed to Q603* | | | | | |  |
| ***603*** | How is the confidentiality maintained in this health facility? | *Please rate out of four for the following statements*(circle that applies) | | | |  |
| *a* | How often are consultations carried out in a manner that protects your confidentiality? | *Never*  *1* | *Sometimes*  *2* | *Usually*  *3* | *Always*  *4* |  |
| *b* | How often is the confidentiality of information provided by you preserved? | *Never*  *1* | *Sometimes*  *2* | *Usually*  *3* | *Always*  *4* |  |
| *c* | How often is the confidentiality of your medical records preserved? | *Never*  *1* | *Sometimes*  *2* | *Usually*  *3* | *Always*  *4* |  |
| *Proceed to Q604*  In the following questions, I will ask you how acceptable the health services are to you and how you feel about the time you spend in the health facility. Please feel free and tell what you feel about each questions. Please answer your ratings with in the response range of **Very Long** to **Very Short**. Very short being the most desired. | | | | | |  |
| ***604*** | How would you rate the waiting time or how quickly you obtained care in/for the following services? | *Please rate the following seven service outlets out of five as indicated below*(circle that applies) | | | | |
|  |  | *Very Long* | *Long* | *Fair* | *Short* | *Very Short* |
| ***a*** | Consultation services | *1* | *2* | *3* | *4* | *5* |
| ***b*** | Emergency services | *1* | *2* | *3* | *4* | *5* |
| ***c*** | Lab services | *1* | *2* | *3* | *4* | *5* |
| ***d*** | Fees/at cashier | *1* | *2* | *3* | *4* | *5* |
| ***e*** | Pharmacy/dispensary | *1* | *2* | *3* | *4* | *5* |
| ***f*** | Reception/card room | *1* | *2* | *3* | *4* | *5* |
| ***g*** | HIV counseling and testing services | *1* | *2* | *3* | *4* | *5* |
| ***h*** | Overall rating of waiting time for HIV/AIDS treatment and care services | *1* | *2* | *3* | *4* | *5* |
| *Proceed to Q605*  In the following questions, I will ask you how acceptable the health services are to you and how you feel about the way you are treated in making choices. Please feel free and tell what you feel about each questions. Please answer your ratings with in the response range of **Never** to **Always**. | | | | | | |
| ***605*** | How often do you have opportunity to make choices about the following in this health facility related to HIV/AIDS treatment and care services? | *Please rate the following three issues out of four as indicated below*  (circle that applies) | | | |  |
| ***a*** | A choice between health care providers in a health care unit | *Never*  *1* | *Sometimes*  *2* | *Usually*  *3* | *Always*  *4* |  |
| ***b*** | A choice between health care units | *Never*  *1* | *Sometimes*  *2* | *Usually*  *3* | *Always*  *4* |  |
| ***c*** | The opportunity to see a specialist/doctor, if you wish to | *Never*  *1* | *Sometimes*  *2* | *Usually*  *3* | *Always*  *4* |  |
| *Proceed to Q606* | | | | | |  |

| *606* | Following are six items that are related to your visits with your doctor. Physicians have different styles in dealing with patients, and we would like to know more about how you have felt about your encounters with your physician. Please rate out of seven i.e. from ‘Strongly disagree’ to ‘Strongly Agree’. | 1  Strongly Disagree | 2  Moderately Disagree | | | | 3  Fairly Disagree | 4  Neutral | 5  Fairly Agree | 6  Moderately Agree | | 7  Strongly Agree |
| --- | --- | --- | --- | --- | --- | --- | --- | --- | --- | --- | --- | --- |
| *a* | I feel that my physician has provided me choices and options | *1* | *2* | | | | *3* | *4* | *5* | *6* | | *7* |
| *b* | I feel understood by my physician | *1* | *2* | | | | *3* | *4* | *5* | *6* | | *7* |
| *c* | My physician conveys confidence in my ability to make changes | *1* | *2* | | | | *3* | *4* | *5* | *6* | | *7* |
| *d* | My physician encourages me to ask questions | *1* | *2* | | | | *3* | *4* | *5* | *6* | | *7* |
| *e* | My physician listens to how I would like to do things | *1* | *2* | | | | *3* | *4* | *5* | *6* | | *7* |
| *f* | My physician tries to understand how I see things before suggesting a new way to do things | *1* | *2* | | | | *3* | *4* | *5* | *6* | | *7* |
| *Proceed to 607* | | | | | | | | | | | | |
| ***607*** | How would you rate your experiences about how well you were treated as human during interactions (communications) with service providers for HIV/AIDS treatment and care services? (Please consider how orientation was provided, your questions were answered, explanations about benefits, risks and options about care plans were provided during your interaction with health workers) | *Please rate the following eight interaction points in this health care facility as indicated below*  (circle that applies) | | | | | | | | | | |
| *a* | Doctors/Other physicians/Specialists | *Very Poor*  *1* | | *Poor*  *2* | | | | *Fair*  *3* | *Good*  *4* | | | *Very Good*  *5* |
| *b* | Nurses | *Very Poor*  *1* | | *Poor*  *2* | | | | *Fair*  *3* | *Good*  *4* | | | *Very Good*  *5* |
| *c* | Interactions with Lab staffs | *Very Poor*  *1* | | *Poor*  *2* | | | | *Fair*  *3* | *Good*  *4* | | | *Very Good*  *5* |
| *d* | Pharmacy/dispensary staffs | *Very Poor*  *1* | | *Poor*  *2* | | | | *Fair*  *3* | *Good*  *4* | | | *Very Good*  *5* |
| *e* | Reception/Card room staffs | *Very Poor*  *1* | | *Poor*  *2* | | | | *Fair*  *3* | *Good*  *4* | | | *Very Good*  *5* |
| *f* | Security staffs | *Very Poor*  *1* | | *Poor*  *2* | | | | *Fair*  *3* | *Good*  *4* | | | *Very Good*  *5* |
| *g* | Other administration staffs (cashers and others) | *Very Poor*  *1* | | *Poor*  *2* | | | | *Fair*  *3* | *Good*  *4* | | | *Very Good*  *5* |
| *h* | How would you rate the overall quality of interaction with staffs of this health facility | *Very Poor*  *1* | | *Poor*  *2* | | | | *Fair*  *3* | *Good*  *4* | | | *Very Good*  *5* |
| ***Proceed to Q608*** | | | | | | | | | | | | |
| ***608*** | How would you rate the financial fairness of the following service outlets to you in this facility? | Please rate the following four types service outlets out of five as indicated below(circle that applies) | | | | | | | | | | |
| *a* | Consultation services (including payment for card) | *Very Unfair*  *1* | | | | *Unfair*  *2* | | *Neutral*  *3* | *Good*  *4* | | *Very Good*  *5* | |
| *b* | Lab services | *Very Unfair*  *1* | | | | *Unfair*  *2* | | *Neutral*  *3* | *Good*  *4* | | *Very Good*  *5* | |
| *c* | Drugs/medical supplies | *Very Unfair*  *1* | | | | *Unfair*  *2* | | *Neutral*  *3* | *Good*  *4* | | *Very Good*  *5* | |
| *d* | Treatment for opportunistic infections | *Very Unfair*  *1* | | | | *Unfair*  *2* | | *Neutral*  *3* | *Good*  *4* | | *Very Good*  *5* | |
| ***Proceed to Q609*** | | | | | | | | | | | | |
| 609 | How would you rate the worthiness of the following expenses relative to services you receive at this health facility? | Please rate the following four questions out of five as indicated below  (circle that applies) | | | | | | | | | | |
| *a* | How would you rate the transportation expenses to health care units? | *Very Unfair*  *1* | | | *Unfair*  *2* | | | *Neutral*  *3* | *Good*  *4* | | | *Very Good*  *5* |
| *b* | How would you rate expenses for food when away for treatment? | *Very Unfair*  *1* | | | *Unfair*  *2* | | | *Neutral*  *3* | *Good*  *4* | | | *Very Good*  *5* |
| *c* | Expenses for accommodation when away for treatment? | *Very Unfair*  *1* | | | *Unfair*  *2* | | | *Neutral*  *3* | *Good*  *4* | | | *Very Good*  *5* |
| *d* | How would you rate the overall financial fairness of the services in the health care units with regard to the quality of care you obtained? | *Very Unfair*  *1* | | | *Unfair*  *2* | | | *Neutral*  *3* | *Good*  *4* | | | *Very Good*  *5* |
| *Proceed to Q701* | | | | | | | | | | | | |

**Perceived Quality of HIV/AIDS Treatment and Care Services**

The following questions are about quality of services. Please tell me how you feel about the quality of services in different units of the health facility.

| 701 | How would you agree or disagree with the following statements regarding how are you treated in this health facility? | Please rate the following three statements out of five as indicated below  (circle that applies) | | | | |
| --- | --- | --- | --- | --- | --- | --- |
| a | The health unit is providing services at the promised time | *Strongly Disagree*  *1* | *Disagree*  *2* | *Neutral*  *3* | *Agree*  *4* | *Strongly Agree*  *5* |
| b | The working hours of the health units are convenient to me | *Strongly Disagree*  *1* | *Disagree*  *2* | *Neutral*  *3* | *Agree*  *4* | *Strongly Agree*  *5* |
| c | Right services are provided to me on the right time | *Strongly Disagree*  *1* | *Disagree*  *2* | *Neutral*  *3* | *Agree*  *4* | *Strongly Agree*  *5* |
| *Proceed to Q702* | | | | | | |
| 702 | How would you agree or disagree with the following statements regarding how is your autonomy respected? | Please rate the following two statements out of five as indicated below  (circle that applies) | | | | |
| a | Employees of the health unit put trust in my ability to understand | *Strongly Disagree*  *1* | *Disagree*  *2* | *Neutral*  *3* | *Agree*  *4* | *Strongly Agree*  *5* |
| b | My demands are served in the health unit | *Strongly Disagree*  *1* | *Disagree*  *2* | *Neutral*  *3* | *Agree*  *4* | *Strongly Agree*  *5* |
| *Proceed to Q703* | | | | | | |
| 703 | How would you agree or disagree the following statements regarding of care providers in this facility? | Please rate the following three statements out of five as indicated below  (circle that applies) | | | | |
| a | Employees have the knowledge to answer my questions related to health care | *Strongly Disagree*  *1* | *Disagree*  *2* | *Neutral*  *3* | *Agree*  *4* | *Strongly Agree*  *5* |
| b | Employees of the health unit are always ready to answer my requests | *Strongly Disagree*  *1* | *Disagree*  *2* | *Neutral*  *3* | *Agree*  *4* | *Strongly Agree*  *5* |
| c | Employees have a neat and professional appearance in the health unit | *Strongly Disagree*  *1* | *Disagree*  *2* | *Neutral*  *3* | *Agree*  *4* | *Strongly Agree*  *5* |
| *Proceed to Q704* | | | | | | |
| 704 | How would you agree or disagree with the following statements based on your experiences of this health facility? | Please rate the following five statements out of five as indicated below  (circle that applies) | | | | |
| a | Patients with similar needs are treated equally in the health unit | *Strongly Disagree*  *1* | *Disagree*  *2* | *Neutral*  *3* | *Agree*  *4* | *Strongly Agree*  *5* |
| b | Patients with unequal needs(severe illness/in pain) are prioritized in the health unit | *Strongly Disagree*  *1* | *Disagree*  *2* | *Neutral*  *3* | *Agree*  *4* | *Strongly Agree*  *5* |
| c | The health unit keeps error-free records of mine | *Strongly Disagree*  *1* | *Disagree*  *2* | *Neutral*  *3* | *Agree*  *4* | *Strongly Agree*  *5* |
| d | The health unit has met my expectations | *Strongly Disagree*  *1* | *Disagree*  *2* | *Neutral*  *3* | *Agree*  *4* | *Strongly Agree*  *5* |
| e | The health unit is providing services as promised | *Strongly Disagree*  *1* | *Disagree*  *2* | *Neutral*  *3* | *Agree*  *4* | *Strongly Agree*  *5* |
| *Proceed to Q705* | | | | | | |
| 705 | How would you rate the overall quality of services in the health units? | *Very Poor*  *1* | *Poor*  *2* | *Neutral*  *3* | *Good*  *4* | *Very Good*  *5* |
| *Proceed to Q801* | | | | | | |

**Satisfaction with HIV/AIDS treatment and care services**

The following questions are about satisfaction with health services. Please tell me how you are satisfied with services in each units.

| 801 | How would you rate your satisfaction or dissatisfaction with HIV/AIDS treatment and care services in this health facility?  (Circle on the corresponding number) | ***Very Dissatisfied***  ***(1)*** | ***Fairly Dissatisfied***  ***(2)*** | ***Neutral***  ***(3)*** | ***Fairly Satisfied***  ***(4)*** | ***Very Satisfied***  ***(5)*** |
| --- | --- | --- | --- | --- | --- | --- |
| a | Overall quality of consultation services | *1* | *2* | *3* | *4* | *5* |
| b | Overall quality of Lab services | *1* | *2* | *3* | *4* | *5* |
| c | Overall quality of the reception | *1* | *2* | *3* | *4* | *5* |
| d | Overall quality of administrative services in the health units | *1* | *2* | *3* | *4* | *5* |
| e | Overall quality of services in the health units relative to your expenses and efforts | *1* | *2* | *3* | *4* | *5* |
| f | Overall quality of the services in the health units? | *1* | *2* | *3* | *4* | *5* |
| *Proceed to Q901* | | | | | | |
