# Supplementary material for: Correlates of perceived access and implications for health system strengthening – lessons from HIV/AIDS treatment and care services in Ethiopia
Source: PLoS One. 2016 Aug 22;11(8):e0161553. doi: 10.1371/journal.pone.0161553 (PMC4993581; doi:10.1371/journal.pone.0161553)
Supplement: S2 File — (DOCX) [file pone.0161553.s002.docx]

| Code | Questions /items | Response | Remark |
| --- | --- | --- | --- |
| 001 | Participant ID (QID in this study) |  |  |
| 002 | ART/pre-ART Enrollment Number |  |  |
| 003 | Name of Healthy facility |  |  |
| 004 | Type of care attended | ART- 1  Pre-ART- 2 |  |
| 005 | Clinical Stage (last) | --------------------------- |  |
| 006 | CD4 Count (last) | --------------------------- |  |
| 007 | Date CD4 count was done (last) | ________________ |  |

S2 File Data Abstraction Form

(to be filled by HIV care provider or unit head)

Questionnaire ID __________

Completed by Name _________________________ Sign ___________________

Date ________________________
