# Supplementary material for: Correction: Correlates of Perceived Access and Implications for Health System Strengthening – Lessons from HIV/AIDS Treatment and Care Services in Ethiopia
Source: PLoS One. 2016 Nov 30;11(11):e0167382. doi: 10.1371/journal.pone.0167382 (PMC5130268; doi:10.1371/journal.pone.0167382)
Supplement: S2 File — (PDF) [file pone.0167382.s002.PDF]

PLOS ONE

RESEARCH ARTICLE

Correlates of perceived access and implications for health system strengthening – lessons from HIV/AIDS treatment and care services in Ethiopia

Bereket Yakob[1,2]*, **Busisiwe Purity Ncama**[1]

1 School of Nursing & Public Health, Howard College, University of KwaZulu-Natal, Durban, South Africa, 2 Health Economics and HIV/AIDS Research Division (HEARD), University of KwaZulu-Natal, Durban, South Africa

* berekbot@yahoo.com

CrossMark
click for updates

OPEN ACCESS

Citation: Yakob B, Ncama BP (2016) Correlates of perceived access and implications for health system strengthening – lessons from HIV/AIDS treatment and care services in Ethiopia. PLoS ONE 11(8): e0161553. doi:10.1371/journal.pone.0161553

Editor: Sunil K Ahuja, South Texas Veterans Health Care System, UNITED STATES

Received: July 5, 2015

Accepted: August 8, 2016

Published: August 22, 2016

Copyright: © 2016 Yakob, Ncama. This is an open access article distributed under the terms of the Creative Commons Attribution License, which permits unrestricted use, distribution, and reproduction in any medium, provided the original author and source are credited.

Data Availability Statement: All relevant data are within the paper and its Supporting Information files.

Funding: The authors received no specific funding for this work.

Competing Interests: The authors have declared that no competing interests exist.

Abstract

Background

Access to healthcare is an important public health concept and has been traditionally measured by using population level parameters, such as availability, distribution and proximity of the health facilities in relation to the population. However, client based factors such as their expectations, experiences and perceptions which impact their evaluations of health care access were not well studied and integrated into health policy frameworks and implementation programs.

Objective

This study aimed to investigate factors associated with perceived access to HIV/AIDS Treatment and care services in Wolaita Zone, Ethiopia.

Methods

A cross-sectional survey was conducted on 492 people living with HIV, with 411 using ART and 81 using pre-ART services accessed at six public sector health facilities from November 2014 to March 2015. Data were analyzed using the ologit function of STATA. The variables explored consisted of socio-demographic and health characteristics, type of health facility, type of care, distance, waiting time, healthcare responsiveness, transportation convenience, satisfaction with service, quality of care, financial fairness, out of pocket expenses and HIV disclosure.

Results

Of the 492 participants, 294 (59.8%) were females and 198 (40.2%) were males, with a mean age of 38.8 years. 23.0% and 12.2% believed they had 'good' or 'very good' access respectively, and 64.8% indicated lower ratings. In the multivariate analysis, distance from the health facility, type of care, HIV clinical stage, out of pocket expenses, employment

status, type of care, HIV disclosure and perceived transportation score were not associated with the perceived access (PA). With a unit increment in satisfaction, perceived quality of care, health system responsiveness, transportation convenience and perceived financial fairness scores, the odds of providing higher rating of PA increased by 29.0% ($p<0.001$), 6.0%($p<0.01$), 100.0% ($p<0.001$), 9.0% ($p<0.05$) and 6.0% ($p<0.05$) respectively.

Conclusion

Perceived quality of care, health system responsiveness, perceived financial fairness, transportation convenience and satisfaction with services were correlates of perceived access and affected healthcare performance. Interventions targeted at improving access to HIV/AIDS treatment and care services should address these factors. Further studies may be needed to confirm the findings.

Introduction and Background

Ensuring that citizens have access to healthcare has become a political goal of many governments [1], and is considered important when measuring health success. In an effort to improve the health of people, governments strive to choose effective health policy frameworks and optimize implementation strategies to ensure universal access to health care [2–5]. The HIV/AIDS pandemic has impacted on health systems in developing countries in particular, and reversed recent gains in health improvement such as reductions in morbidity and mortality and quality of care by putting additional pressure on already overstretched resources due to its associated complications and co-infections [4, 6]. As a result of this additional pressure, more effective, better equipped, better staffed and well organized health facilities are required, improve the quality of life of people infected with HIV and to prevent and alleviate the health consequences of morbidity and mortality [3, 4, 7].

In response to the HIV/AIDS pandemic, several interventions have been implemented at global and country levels to mitigate its impact and spearhead infection prevention and control activities [3–5, 7, 8]. Despite these efforts, it has claimed over 39 million lives, and caused pain and suffering among those affected and afflicted by the virus [5, 9]. The efforts have however assisted in reducing the scope of the pandemic, and there are signs of stabilization, with reductions in new infections and deaths due to AIDS having been observed in many countries. Providing universal access to antiretroviral therapy (ART) has become an important part of combating the disease[4, 7, 10], with recent estimations indicating that approximately 15 million (40%) of the 36.9 million people living with HIV using ART in 2015 [11, 12]. Within this global context,70% of people infected with HIV and 73% of all deaths due to AIDS were in the sub-Saharan Africa (SSA), with60% of those infected not having access to ART in this region [9, 12].

Ethiopia had over 800,000 people infected with HIV in 2014, with 43.0% reportedly having access to ART [13, 14]. Despite the successful scale up of ART since 2005 [5], only 70.3% of people who ‘ever started on ART’ remained on treatment in 2013, indicating problems of access and retention [10, 15]. The low access to HIV/AIDS treatment and care services (HATCS) in the country was due to a number of factors, including low service coverage, high dropout from HIV care, adherence problems, low services utilization, and poor quality and acceptability of health services [16–19], all indicating that service delivery performances fell short of the desired level [20].

Access to healthcare is an important public health concept [21] and has been traditionally measured by using population level parameters, such as availability, distribution and proximity of the health facilities in relation to the population [22–25]. This approach has not accounted for the soft but important client-based factors such as the health status and mind-set of clients, perceptions, expectations and experiences and the socio-cultural factors (norms, belief systems and accepted behaviors) that affect people, all of which enable or prohibit access to care [26–31].

The environments in which clients are treated have been referred to as the responsiveness of the health care system and have not been well studied [32]. It has eight domains regarding how health systems handle the legitimate expectations of clients, is measured by eight domains such as respect (care with dignity), choice (of care provider and care units), confidentiality, prompt attention (waiting time and responding to client calls for help), autonomy (involvement in medical decision making), communication (providing information and interaction), amenities of care (facilities and infrastructure) and access to social support (family, prayer, etc.) [32]. When these client-based factors are not satisfied, the desired outcome of the healthcare cannot be achieved or will falter [33].

Clients' perceptions of how and in what conditions they are treated impacts on their evaluation of the overall quality of care, responsiveness of the health facilities and satisfaction with services [20, 32, 34]. However, the relationships between the responsiveness of healthcare, financial fairness, perceived quality of care and clients' satisfaction with care and perceived access to care have not been extensively studied. Failing to satisfy clients has grave impacts on outcomes of clinical care such as poor adherence and retention in HIV/AIDS care, and ultimately results in treatment failure or fail to achieve viral suppression. This study therefore aimed to investigate the correlates of perceived access (PA) to HIV/AIDS treatment and care services (HATCS) in Wolaita Zone, southern Ethiopia.

Methods

Defining Access

Over the years, policy makers, scholars, and clients had used a number of definitions and concepts of access to healthcare [33, 35–40]. Affordability and distribution of the healthcare facilities have interested policy makers, while social scholars stressed the social distance from service outlets and their comprehensiveness in terms of the socio-economic, political and cultural dimensions [26, 35, 41]. The clients, as consumers of the health system and part of the society, have their own ways of feeling and understanding about how accessible the services are to them or not [33, 41, 42]. Clients' views extend to the responsiveness, acceptability, quality, equality and continuity of care and not only the physical access to healthcare [33, 43].

After reviewing several conceptual definitions and frameworks presented by scholars such as Donabedian [33], Penchesky and Thomas [36], Aday and Andersen (41), McIntyre [39] and Institute of Medicine [44],access was operationalized as follows. Healthcare access is the extent the health system fits, inhibits or initiates the willingness and ability of individuals to enter to, to receive and benefit from the outcomes of, and to get satisfaction from the services. It is the process of knowing about, seeking, entering, passing through, getting satisfaction from the care and benefiting from the outcomes of health service, and it is not merely having consultation with the health care provider and/or getting prescriptions. How clients perceive these processes has been designated as 'perceived access' in this study.

The Study Area

This study was conducted in the Wolaita Zone of the Southern Nations, Nationalities and Peoples Region (SNNPR) of Ethiopia, which is approximately 162 km southwest of the regional

capital Hawassa, and 330km south of the national capital Addis Ababa. It has a total area of 4,471.3 km^2, and with 1,866,400 inhabitants, is one of the most densely populated areas in the country, with an average of 385people/km^2 in 2014 (projections based on the 2007 Population and Housing Census of Ethiopia). It was the second largest populated zone in the region and on average 4.8 people lived in each household. According to the2007 census, 98.0% of the population were Christians, 96.8% spoke Wolaita Donna,46.1% were educated (literate) and 97.2% were employed, mainly in agriculture [45].

The topography of the zone ranges from hilly terrain to flat lowlands, with varying climatic conditions. The zone had 12 woredas (districts) and three town administrations, with 324 kebeles (an equivalent of villages). Recently, the potential health services coverage (people who have physical access to healthcare) of the zone increased to over 95.0% with the scale up of existing health posts. There were three hospitals, 63 health centers, 333 health posts, and several private clinics and drug venders. A total of 2,035 health professionals and over 701 health extension workers delivered health services in the zone in 2013 [46]. The zone was selected for the study as a result of the large number of estimated people infected with HIV (16,795 people), this being a function of its population size based on the regional HIV prevalence rate of 0.9%, lower ART utilization rate and the presence of many high risk corridors (center for business and transactions, cash crops and mega projects hiring many people, etc.). This study was part of a bigger study and the information contained in the study area has been published elsewhere [47, 48].

Study Design, Period and Sampling

An analytic cross-sectional study was conducted in six health facilities in Wolaita Zone from November 1, 2014 to March 15, 2015. The study population was all people infected with HIV and the sampling frame was all people using HIV treatment and care services during the study period in Wolaita Zone. Of three hospitals and 63 health centers, only 14 had started delivering ART services by 2013, eight health facilities only having started to provide them in 2014 or later were excluded. As a result, six public sector health facilities (1 hospital and 5 health centers) which started both pre-ART and ART services by 2013 were chosen as they accounted for 74.6% of all people using ART care (2262 out of 3038 clients) in 2014 in the zone (the names of the health facilities participated in the study were not displayed due to confidentiality reasons).

All people living with HIV who were using pre-ART and ART services were considered for the study. As the number of clients using pre-ART services at each facility was very small, all those coming for the service were invited to participate. However, there were a relatively large number of potential participants on ART which necessitated the use of sampling technique to identify those who would be included. Depending on the number of clients each facility had, the sample size was initially determined proportionally. Eligible clients were then selected randomly using the ART client registration number (excluding those who were newly enrolling during the study period, transferred out, lost to follow up, dropped care or died). The sample size was determined by using the formula for one-sample population proportion for analytic methods [49]

$$\boldsymbol{n} = \frac{\boldsymbol{\pi}(\mathbf{1}-\boldsymbol{\pi})[\boldsymbol{Z}\mathbf{1}-\boldsymbol{\alpha}/\mathbf{2}]^{\mathbf{2}}}{(\boldsymbol{SE})^{\mathbf{2}}}$$

n is the sample size required, $z_{1-}\alpha/2$ is the test statistic at 95% confidence level for two-sided test, π is the proportion of population affected by the health phenomena being studied. The commonly used value for *Type I error* (α) 0.05 was used, while power of the study (1-β) was estimated at 80% [49]. Due to the difficulty of finding studies on perceived access to HATCS in Ethiopia that could advise on sample size calculation, an estimated prevalence of 50.0% of

perceived access was used which provided the largest sample size possible. The calculation indicated a sample size of 385 clients, with a 10.0% contingency being added for assumed non-response, making the total sample size for ART clients 424.

The PI randomly selected potential participants (ART clients) using the ART client registration numbers presented by the focal persons of the ART units. Thereafter, the selected registration numbers were matched with the random numbers and forwarded to ART clinic staffs. After being oriented and giving consent, potential ART clients were connected with the trained data collectors by the HIV care unit staffs on their routine appointment dates for refilling ART drugs. The information regarding study design and sampling has also been published elsewhere [47, 48].

Inclusion and Exclusion Criteria

Being a minor (age < 18 year) and/or admitted for inpatient care, refusal to participate and/or not wanting to sign the consent form (11 people), and those from other zones (than the study area) constituted exclusion criteria. Clients coming from other zones to obtain HATCS were excluded to reduce contamination of information with knowledge and experiences. All clients living with HIV, aged 18 year or above, who lived in Wolaita Zone for at least six months during the study and attended HATCS in the selected facilities, gave consent to participate and who were not seriously ill (not in inpatient care) were invited and included.

Instrument and Variables of the Study

The questionnaire (S1 File) was adapted from validated instruments of the WHO's responsiveness domains that were used in multi-country studies [50–52], the Patient Health Questionnaire (PHQ9), the SERVQUAL scale[53, 54], and Lej and Jolibert (2012) study [55]. The contents of the questionnaire included clinical data abstraction form (HIV clinical stage, most recent CD4 count and type of care) (S2 File), socio-demographic characteristics, utilization of HATCS (length of using HATCS and use of tuberculosis treatment services), own health assessments, number of years lived with HIV, HIV status disclosure (Yes/No) and stigma (Yes/No), PHQ-9 scales for probable mental depression assessment, geospatial factors (distance, difficulty of the landscape and time required to travel to health facilities), satisfaction with care, transportation availability, domains of health system responsiveness and financial fairness of the services and out of pocket expenses.

Perceived access was the dependent variable of the study. Participants were asked to rate how they felt about the overall access to HATCS taking into consideration their experiences of the availability of care, distance from the health facility and transportation convenience, time spent on travel and waiting time, the way they were treated and the quality of care i.e. rated from 'very bad' to 'very good'. All other variables mentioned were the independent variables the study.

To measure perceived quality of HATCS, a scale was adapted from six items of the SERVQUAL scale [53, 54] and six items of Lej and Jolibert (2012) [55] study and two items (about medical record keeping and impartial treatment) added after reviewing literature. Accordingly, the total perceived quality of care score was computed from 13 questions (excluding the overall perceived quality of care rating to avoid overlap)that focused on providing service as promised on the anticipated time, provider expertise, equality and equity of services, and regard for clients' interest and ability, each rated out of five from 'very poor' to 'very good'.

A long key-informant interview instrument of the WHO multi-country health systems responsiveness study was reviewed and items related to its eight domains were selected, and further checked for its appropriateness for the Ethiopian context and local clinical practice.

This was augmented by incorporating six items adapted from the Health Care Climate Questionnaire short form (autonomy) [56]. The responsiveness score was computed from the relevant domain questions, such as interaction with people involved in health service delivery (8 questions), autonomy (16 questions), amenities of care (11 questions), confidentiality and privacy (3 questions), respect and dignity for clients (7 questions) and prompt attention (7 questions). The internal consistency of the responsiveness scale was measured with Cronbach's alpha which stood at 0.88 (very good consistency) (Yakob and Ncama, manuscript submitted).

Perceived transportation convenience score was adapted from four items of the WHOQOL full questionnaire (F23.1 –F23.4)[57] and a score was computed. The items were questions about client's satisfaction with transportation convenience and about how difficult or easy it was to get transport to the health facilities i.e. rated from 'very difficult' to 'very easy'. The perceived financial fairness score was computed from eight questions developed after literature review about how fair expenses were at each of the care units and the relative worth of the care when the expenses were taken into account, rated from 'very unfair' to 'very fair'.

The instrument included 7 questions about how clients were satisfied with services in each of service outlets (6 questions) and overall satisfaction with care (1 question). An item adapted from Lej and Jolibert (2012) study i.e. 'Overall, how satisfied are you with your hospital?' was extended for six service outlets involved in HIV care. Total satisfaction score was computed from the six questions about how clients were satisfied at each services outlets i.e. rated from 'very dissatisfied' to 'very satisfied'. All the questions were presented in a Likert Scale including a 'neutral' category in the middle.

The instrument was prepared in English and translated into Wolaita and Amharic (local languages) by experts and back translated to English until consistency was reached. It was piloted on 20 people using HATCS in one of the hospitals not selected for the study. Based on the field observations, few questions were rephrased while 'access to social support' domain of responsiveness was removed after consultation with experts and literature as it was less applicable in outpatient care. Although pre-study psychometric testing was not undertaken, the instrument was presented, scrutinized and corrected by experts in the field.

Data Collection

Nine data collectors (five females and 4 four males) who had health qualification (at least Diploma in Nursing or BSc in Public Health Officer) were fluent in both Wolaita Dona and Amharic were identified from the zone. The data collectors were neither staffs of nor affiliated to the health facilities to which they were assigned to collect data. A research assistant with a Master's in Public Health and research experience was hired, and supervised data collection together with the principal investigator. A three days training session was conducted for data collectors and research assistant on research ethics, interviewing techniques and the data collection instruments.

Data Analysis

Data from the completed hard copy questionnaires were entered into EPI/INFO v 7, cleaned, prepared, and transformed i.e. recoded, merged, split and computed, to create new variables in preparation for further analysis with STATA 13.1 (StataCorp, Texas, USA). For the statistical significance and estimations, 95.0% confidence interval (CI) and p-value <0.05 were used. In bivariate and multivariate analysis, ordinal logistic regression was chosen due to the ordinal nature of the outcome variable i.e. perceived access (PA), which was rated by the respondents as 'very poor', 'poor', 'neutral', 'good' and 'very good'.

Using this rank-ordered outcome variable, bivariate and multivariate ordinal logistic regressions were conducted. As 'proportional odds ratio' (cumulative probability model) was used in the ordinal logistic regression (*ologit*), only four cut-off points (COP) or thresholds were possible for the five response of the PA, and the odds ratios were assumed equal across the COPs. Proportional odds ratio enables the estimation of cumulative probability of being at or below a given response category of the ordinal dependent variable[58, 59], PA in this study. For simplicity of interpretation and understanding, the possible COPs of the PA were designated as follows: COP 1 was a cumulative probability of being at or below 'poor' rating; COP 2 was at or below a 'neutral' rating; COP 3 was a cumulative probability of being at or below 'good' rating; and COP 4 was a cumulative probability of being at or below 'very good' rating. A leap from a lower response category to a subsequent higher category, such as from COP 1 to COP 2, was considered a positive condition, while the reverse was an opposite condition on account of the perceived access to HATCS in this study.

The independent variables, which showed $p \geq 0.10$ in the bivariate analysis, were not considered in further analysis, while all other variables with $p \leq 0.10$, such as the type of health facility, type of care registered for, HIV clinical stage, years lived with HIV, transportation convenience score, satisfaction score, perceived financial fairness score, perceived quality score and responsiveness score, were included in multivariate analysis. They were fitted to the ordinal logistic regression model in STATA 13.1 (*ologit* function) where they were tested for interactions and multicollinearity.

The proportional odds ratio model assumptions were checked and none were violated i.e. the model fitting showed $p < 0.001$ in *ologit* function; Brant's test of parallel lines showed $p > 0.05$ using '*brant*, *detail*' function; and the approximate likelihood ratio test of proportionality of odds across response categories showed $p > 0.05$ using the *omodel logit* function. When fitted, the *ologit* model did not show any significant collinearity and did not remove any of the variables entered. This was further checked by the *fit* and *vif (variable inflation factor)* functions, the results of the *vif* being below five. In addition, during a full factorial analysis, only trivial (non-significant) interaction effects were observed and were ignored, and only the main effects were considered in further analysis.

Ethical Considerations

The study proposal was approved by the Biomedical Research Ethics Committee (BREC) of the University of KwaZulu-Natal (South Africa) and Wolaita Soddo University Institutional Review Board (Soddo, Ethiopia). Upon receiving clearance, permission letters were obtained from Wolaita Zone Chief Administrator's Office, Wolaita Zone Health Department and all respective district health offices and health facilities.

The data collectors read the information sheet and consent form, and obtained assent from all participants before the interview. When the participants were uneducated, witnesses (who were relatives/friends) accompanying the potential participant who could read and write, were asked to sign for them. Those who refused to participate, mainly due to inconvenience or any reason they did not want to mention were excluded. The study participants were provided with tea/coffee plus snacks during the interview to make the stay relaxing and attractive. Upon completion, the consent forms were detached from the questionnaire and kept in a locked cabinet. Data were entered into a desktop computer owned by the PI that was password protected.

The identifying information of the participants were not collected, with their codes, which were only known by the PI and ART clinical staffs, being used to maintain confidentiality and privacy. In addition, the data collectors signed non-disclosure agreement form. The study

findings will be made available to the participants, program managers and interested researchers via different channels i.e. publication, reports, and conference presentations.

Results

The results section has been presented as the socio-demographics characteristics, health characteristics and factors associated with access to HATCS. Among the participants, 266(54.1%) and 226(45.9%) were obtaining HATCS at the hospital and health centers, respectively.

The socio-demographic Characteristics

A total of 492 people living with HIV participated in the study, of whom 83.5% were on ART and 16.5% on pre-ART, providing a response rate of96.9%for people on ART (out of 424 approached). The response rate for people on pre-ART care could not be computed as all of those visiting the HIV clinics were invited to participate in the study. The participants had a mean age of 38.78 years with a standard deviation equals to 8.81 years (minimum and maximum ages were18 and 71 years). Three people in inpatient care and four people who failed to consent were excluded, as were six people who could not be traced. All those consented to participate completed the interview. On average, since knowing HIV status, the participants had lived with HIV for 5.89 years with a standard deviation equals to 2.87 years (ranging from one month to16 years), the majority being urban residents (68.5%) and women (59.8%). Participants earned 594.57ETB (equivalent to US$30), a monthly income of nil to 4725 ETB (US $236). Details about the socio-demographic characteristics are shown in Table 1.

Health Characteristics

As shown in Table 2, the majority of participants was on HIV Clinical Stage 1 (72.8%) and was registered for ART (83.5%). Approximately, 44.7% had a CD4 count of less than 500/cm^3, 22.3% were co-infected with TB, and 16.3% had a probable mild to major depression based on the PHQ 9 scores.

Factors associated with HATCS

According to the estimations by the participants, on average, the health facilities were located 16.7±13.6km from their houses, with 212(43.4%) living 5–10km away, 114 (23.4%) living 11–20 km, and 162(33.2%) living >20km further from the health facilities. In addition, when they were asked about the difficulty of the landscape on the way to the healthcare facility, 34(7.0%) indicated very difficult, 103 (21.1%) difficult, 145 (29.8%) neutral, 155 (31.8%) easy and 50 (10.3%) very easy. When asked where they first sought HIV care, 192 (39.6%) went to traditional healing services (herbal, prayer and/ or holy water services). With regard to the means of transportation, 155(32.5%) walked on foot while 322(67.5%) used taxis or buses. As shown in Fig 1, each time the clients left their houses for HATCS, an average of 2.23±1.62 hours (ranging from 40 minutes to 24 hours) were spent getting to and from the health facility and obtaining care.

The participants were asked to rate the overall access to HATCS (perceived access) based on their perceptions, with their responses indicated in Fig 2. Approximately 23.1% and 12.2% perceived 'good' and 'very good' access, respectively, while the remainder believed they had either 'neutral', 'poor' or 'very poor' access.

Bivariate analyses were conducted with the socio-demographic characteristics such as age group, sex, residence, marital status, religion, employment status, perceived family income and number of people in a household. In addition, bivariate analysis was carried with the health status characteristics of the respondents such as type of care (ART/pre-ART), HIV clinical staging

Table 1. Socio-demographic characteristics of participants, Wolaita Zone, Ethiopia, 2015.

Variables	Number	%
Age Group		
18–24 year	31	6.3
25–34 year	213	43.3
35–44 year	167	33.9
45+ year	81	16.5
Educational status		
No formal education	63	12.8
Only basic formal education (read & write)	29	5.9
Primary (Grade 1–4)	74	15.0
Primary (Grade 5–8)	155	31.5
High School (Grade 9 –Preparatory)	110	22.4
Vocational Training (after high school)	16	3.3
College/University	45	9.1
Marital status		
Never Married	40	8.1
Currently Married	334	67.9
Divorced	52	10.6
Widowed	48	9.8
Separated	13	2.6
Cohabiting	3	0.6
Don't want to mention	2	0.4
Employment status		
Paid work	106	21.7
Own business (including farming)	246	50.4
Student	33	6.8
Retired	15	3.1
Unemployed	88	18.0
Average Monthly Income		
Range	0-4725ETB*	
Average	594.57ETB*	
Perceived Sufficiency of Income		
Low	345	70.1
Medium	140	28.5
High	7	1.4
Religion		
Orthodox Christian	189	38.7
Protestant Christian	275	56.4
Catholic	13	2.6
Muslim	8	1.6

*Ethiopian Birr (currency).

doi:10.1371/journal.pone.0161553.t001

and CD4 count and depression. It was also conducted with other variables such as the means of transportation used, disclosure of HIV sero-status, time spent to obtain HATCS (in hours), out of pocket expenses, perceived quality of care score, perceived responsiveness score, perceived transportation facility accessibility score, satisfaction score and perceived financial fairness score.

Table 2. Health Characteristics of the Participants, Wolaita Zone, 2015.

Health Characteristics	Number	%
Type of HIV care		
On pre-ART	81	16.5
On ART	411	83.5
Clinical Stage		
Stage 1	358	72.8
Stage 2	107	21.7
Stage 3	27	5.5
CD4 Count (most recent)		
<500/cm3	212	44.7
500+/cm33	262	55.3
Lived with HIV		
Minimum—Maximum	1 month –16 years	
Median	6.00 years	
*Mean±SD***	5.89±2.87 years	
PHQ 9* Score		
< 5 *(no depression)*	410	83.7
$\geq$ 5 *(depression)*	80	16.3
Co-infected with TB		
Yes	108	22.3
No	377	77.7
Disclosed HIV Status		
Yes	412	84.4
No	76	15.6

*Patient health questionnaire (9 item);

** SD–standard deviation.

doi:10.1371/journal.pone.0161553.t002

In bivariate analyses (Table 3), only distance from the health facility, type of health facility, type of care, HIV clinical stage, total satisfaction score, perceived financial fairness score, perceived quality and responsiveness score showed statistically significant association with perceived access to HATCS ($p<0.05$), and are discussed below.

The outputs of multivariate analysis (also displayed in Table 3) showed that the distance from health facilities, HIV clinical stage, out of pocket expenses, type of care and type of health facility were not significantly associated with PA. However, the satisfaction, perceived quality of care, responsiveness, financial fairness and perceived transportation convenience scores showed significant association with the PA.

In the multivariate analysis, with regard to the perceived quality, the clients with higher score were more likely to provide higher rating of perceived access to HATCS ($p<0.01$) in the COPs. In other words, if other conditions were kept constant, a unit increase in the perceived quality score would result in a 6.0% increase in the odds of providing higher category rating of PA in the COPs ($p<0.01$). The clients with higher satisfaction score were more likely to provide higher rating of PA ($p<0.001$) i.e. when other conditions were kept constant, a unit increase in the satisfaction score resulted in a 29.0% increase in the odds of providing higher rating of PA. In the same manner, a unit increase in the responsiveness score would result in a 10.0% increase in the likelihood of higher category rating of PA of HATCS in the COPs ($p<0.001$). Increments in the transportation convenience and financial fairness scores were also associated

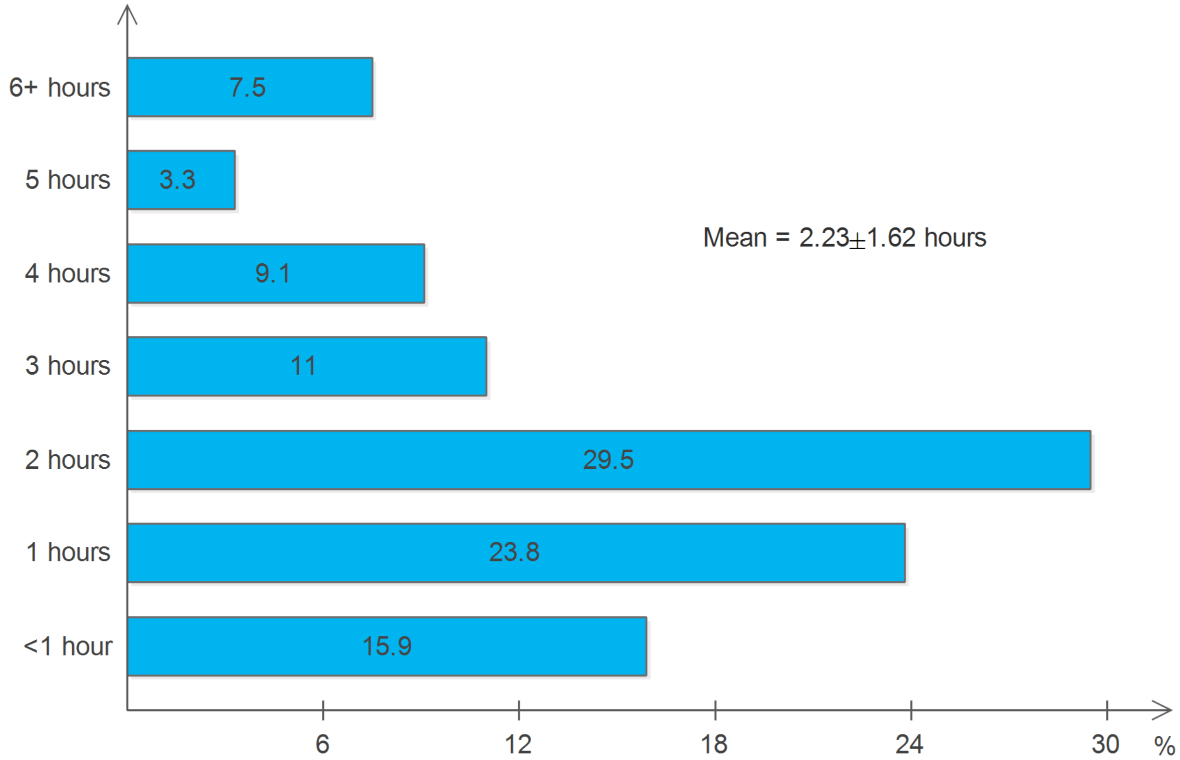

Fig 1. Number of hours spent to obtain care as reported by the participants.

doi:10.1371/journal.pone.0161553.g001

with 9.0% ($p<0.05$) and 10.0% ($p<0.01$) increases in the odds of providing higher rating of the PA, respectively, if other conditions were kept constant.

Discussion

In this study, the PA to HATCS, as opposed to the common objective approaches such as spatial access [23, 25] was assessed. The spatial access approaches rely heavily on objective measurements, such as the coverage and distribution of healthcare facilities and the utilization of services [60, 61], which mainly describe the population level access to care essential to addressing the availability of health facilities and reach [23, 24, 62]. Without undermining the importance of the spatial access to care, the key aspects of the non-spatial patient-based dimensions of access [63]are discussed below. This study has explored the qualitative valuations of access to care by the users or intended users as important measures [30, 43, 64, 65]. This study demonstrated how these qualitative factors could be parametrized and to what extent they affected access to HATCS in the following subsections.

Health Characteristics

The findings of the study showed no significant associations between health characteristics of the clients such as HIV co-infection with TB, CD4 count, years lived with HIV and mental depression, and the PA to HATCS. The study also demonstrated that the satisfaction with care relative to other factors was a highly influential factor in PA ($p<0.001$), which could be affected by the health characteristics. Further analysis of the data from the survey published elsewhere showed that satisfaction with care was negatively affected by the presence of probable mental depression [66]. Similarly, evidences from other studies showed that the health status of clients

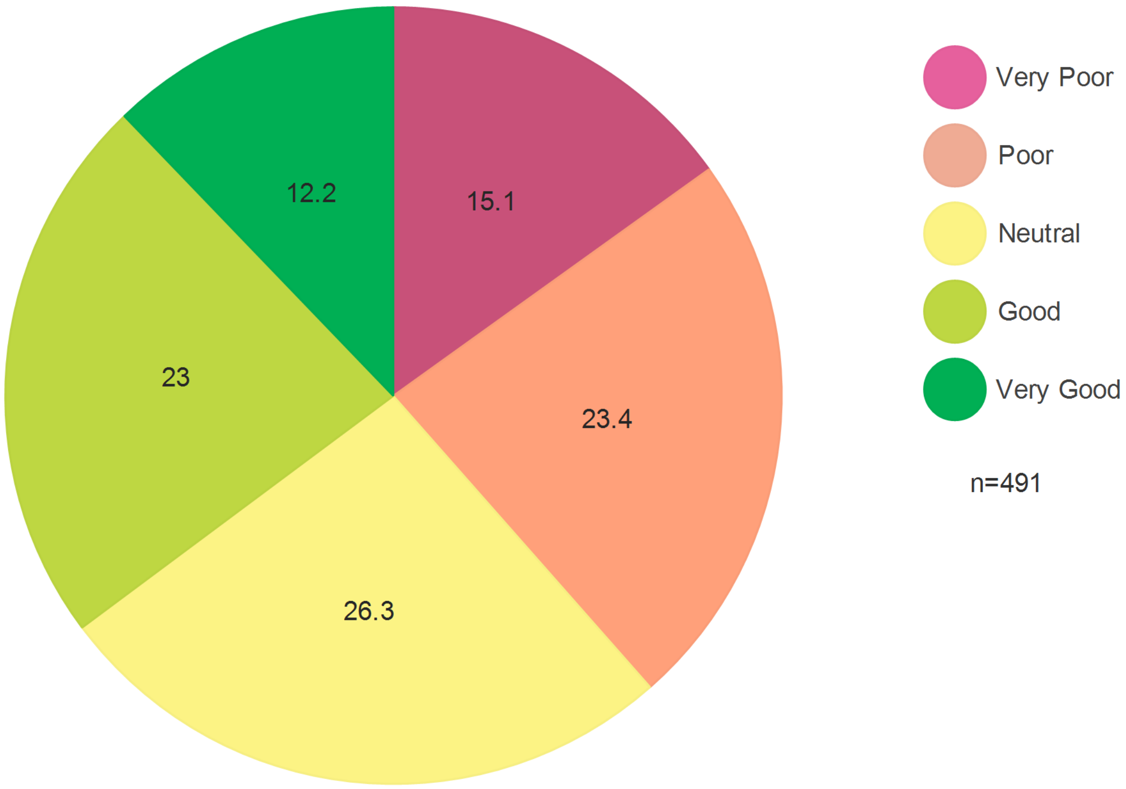

Fig 2. Perceived access to HIV/AIDS treatment and care services as rated by the participants (%).

doi:10.1371/journal.pone.0161553.g002

impacts on the satisfaction with care [67–69], which is an important outcome of access [26, 41]. In addition, it impacts HIV/AIDS treatment adherence and outcomes, and the quality of life of people living with the disease [68, 70–73]. The quality of life and health status of clients were reported to influence the satisfaction with life [74] that affects how people perceive and evaluate things important to them, including medical care [26, 33]. Healthcare facilities were, therefore, expected to satisfy those clients presenting with these interlacing factors, which could be challenging and demanding. Studies need to be conducted to demonstrate how health characteristics of clients would influence satisfaction with the services obtained and to establish their intentions to revisit the health facility in the future.

Experiential, Perceived and Healthcare Factors

In this study, out-of-pocket expense was not significantly associated with the PA, showing that it was not the money spent on and for care did not matter while how fair the expenses were in relation to the services (perceived financial fairness)influenced PA. This was supported by the positive relationships demonstrated between the financial fairness score and PA when adjusted for other factors ($p<0.01$). A further analysis of the data from the same survey indicated that perceived financial fairness influenced perceived quality of care [75] indicating its influence on PA. This study also showed that the perceived financial fairness was as important as the perceived quality of care in predicting the perceived access to HATCS. The clients appeared to weigh the worthiness of access in respect of its totality such as quality, responsiveness, satisfaction with care and transportation convenience rather than merely the amount of out of pocket expenses incurred for the services. In this regard, several studies reported the different effects

Table 3. Bivariate and Multivariate Analysis of Factors Associated with Perceived Access to HATCS, Wolaita Zone (Ordinal Logistic Regression Results).

Variables	Crude OR (95% CI)	Adjusted OR (95% CI)
Distance from Health facility (km)	1.01[1.01–1.03]*	1.00[0.98–1.02]
Type of Health Facility		
Health Center	2.52[1.81–3.50]***	0.86[0.51–1.44]
Hospital	1.00	1.00
HIV Clinical Stage		
Stage 1	1.00	1.00
Stage 2	0.63[0.43–0.93]*	1.26[0.75–2.11]
Stage 3	0.54[0.27–1.12]	0.24[0.08–0.67]
Years lived with HIV	0.94[0.89–0.99]*	0.98[0.90–1.05]
Out of Pocket Expenses		
Yes	0.61[0.41–0.91]*	1.63[0.92–2.90]
No	1.00	1.00
Type of Care Registered for		
ART	0.37[0.24–0.57]***	0.78[0.43–1.44]
Pre-ART	1.00	1.00
Total Satisfaction Score	1.67[1.55–1.80]***	1.29[1.16–1.45]***
Perceived Financial Fairness Score	1.11[1.07–1.15]***	1.06[1.02–1.10]**
Perceived Quality of Care Score	1.16[1.13–1.18]***	1.06[1.03–1.10]**
Total Responsiveness Score	1.12[1.10–1.14]***	1.10[1.08–1.12]***
Perceived Transportation Convenience Score	1.09[1.02–1.17]*	1.09[1.01–1.20]*

*p<0.05;
** p<0.01;
*** p<0.001;
CI–confidence interval;
OR–odds ratio.

doi:10.1371/journal.pone.0161553.t003

of out of pocket expenses that impeded the utilization of health services in different circumstances by delaying care seeking, limiting entrance to healthcare or increasing the discontent with services, especially affecting the poor [76–78]. User fees for health care in developing countries was found to affect adherence to treatments [79], while better interactions between the clients and care providers compensated the negative impacts of out of pocket expenses in some settings [43].

Although the findings of this study did not support the distance from health facility, hours spent in transit to obtain care and waiting time and means of transportation as associated factors to the PA, it is worth noting the potential impact these factors might have on the satisfaction with care given. For instance, a third of the participants travelled through difficult landscapes and a third walked and spent over two hours on average to obtain HATCS. The study also showed, the transportation convenience was found to be an important factor in evaluating the PA, indicating the influence of the availability of, challenges and satisfaction with transportation facilities in access to HATCS. A qualitative study conducted alongside this study showed that unavailability of HIV care centers in proximity to clients, lack of money to pay for transportation, food and accommodation, and food insecurity resulted in quitting ART and loss to follow up [80]. Across the *continuum of care*, health facilities differ, depending on the type, level and quality of care they offer [81], in addition to how far they are located relative to their clients[60, 61] and the waiting time for services. The clients perceive these factors

discriminately, and make judgments about how the care should be, and to what extent the health facilities had met their expectations [33, 82]. Improving access to transportation facilities, locating health facilities providing HATCS near to the clients and reducing their waiting time might improve satisfaction with services, which needs further studies to establish the relationships with these factors.

The study showed that with improvements in perceived quality of care, healthcare responsiveness, perceived financial fairness, satisfaction with services and perceived transportation convenience, the clients of HATCS were more likely to provide higher rating of PA. The individual level assessments of quality of care appeared to be a good measure of the degrees of fit between the health facilities and clients' expectations, and could indicate important areas to focus in intervention programs to improve access to healthcare. Although it was difficult to make comparisons with studies on access conducted elsewhere [30, 43, 82–84], due to the differences in the objectives and methods used and how access was defined, this study showed the five factors that were correlated and played significant roles in the PA to HATCS. Based on the variables involved in the computation of perceived quality of care, improvements in the technical expertise/professionalism, delivering services as promised, ensuring respect for clients by trusting in their ability to understand and improve their health, and maintaining equity and equality of care might assist in increasing the perceived quality of HATCS.

The study also showed that an increase in the responsiveness of the care resulted in higher rating of the PA of HATCS, indicating the important role it played in the clients' perceptions regarding access. As responsiveness is a composite parameter of several non-medical aspects of care, such as autonomy, client-provider interactions, respect for clients and dignity, amenities to care, access to social support, and orientation [34, 85, 86],the results might indicate the importance of each care facility evaluating the way, where and how care is delivered to clients. Satisfaction with services has been considered a proxy indicator of access to care and a measure of the outcome of care, as reported by several studies [26, 30, 41, 83, 87]. Despite the low regard given by many health facilities to the clients' valuation of care [33], this study found the satisfaction with services, which is inherent in the clients, to be an important factor affecting PA i.e. a unit increase contributing 29.0% improvement in the rating of perceived access across the levels of the evaluation categories (COPs).

Based on the findings, improving the quality of care, responsiveness, financial fairness, transportation convenience and clients' satisfaction with the services should be the prime objective for any health facility providing HATCS that claims to be client-centered or people-centered.

Limitations, Conclusion and Recommendations

Despite the strengths of the study such as a large sample size, being based on primary data and using innovative ways to compute scores for perceived quality, satisfaction, responsiveness, financial fairness and transportation convenience, the study also had limitations. These were the cross-sectional nature of the study, making the establishment of causal relationships difficult, and the fact that the results might not be generalizable beyond the study area, requiring further studies in other areas to do so. The study was conducted inside health facilities due to administrative issues that might have slightly or favorably biased the responses by interviewees. The study was conducted on people already accessing some form of HIV care (ART/ pre-ART), generalizing the study findings to those not utilizing might be difficult. Further studies need to be conducted to do so. If conducted, structural equation modeling would have assisted in establishing the paths and defining the relationships between PA and its components such as perceived quality of care, satisfaction with care, financial fairness, transportation convenience and health characteristics of the clients.

Acknowledging the above limitations, the study has brought to light important evidences regarding the factors associated with perceived access to HATCS. Perceived quality of care, responsiveness, satisfaction with care, financial fairness and transportation convenience were found to be the factors associated with of perceived access, satisfaction with services being the most important factor. Based on these findings, the authors recommend increased attention to satisfying clients' expectations and needs in order to gain confidence from and to be recognized as better performing sites of HATCS. The results of the study could be cautiously generalized to similar contexts if similar methods were applied. For wider applicability, and to demonstrate the relationships between the health characteristics of the clients and satisfaction with services, responsiveness and perceived quality of care, further studies might be required.

Supporting Information

S1 File. Questionnaire.
(DOCX)

S2 File. Clinic Data Abstraction Form.
(DOCX)

Acknowledgments

The authors thank all participants for offering their time and providing the information required for the study. The authors extend their acknowledegements to Wolaita Zone Health Department, Boditti Town Administration Health Unit, Humbo Tebela Woreda Health Office, Areka Town Administration Health Unit, Soddo Town Administration Health Unit, Ofa Woreda Health Office and all health facilities for providing permission and support during the study. Many thanks to the data collectors, data entry clerk and to all those contributed to this study. Finally, the authors acknowledge HEARD and the College of Health Sciences, School of Nursing and Public Health, Public Health Medicine of the University of KwaZulu–Natal for providing learning environment. All literatures used in this article are acknowledged.

Author Contributions

Conceived and designed the experiments: BY BPN.

Performed the experiments: BY.

Analyzed the data: BY.

Contributed reagents/materials/analysis tools: BY BPN.

Wrote the paper: BY BPN.

Supervised data analysis: BPN.

References

1. Wagner AK, Graves AJ, Reiss SK, LeCates R, Zhang F, Ross-Degnan D. Access to care and medicines, burden of health care expenditures, and risk protection: Results from the World Health Survey. Health Policy. 2011; 100(2–3):151–8. doi: 10.1016/j.healthpol.2010.08.004 PMID: 20828854
2. Kendall AE. U.S. Response to the Global Threat of HIV/AIDS: Basic Facts CRS Report for Congress, 2012.
3. Dionisio D, Esperti F, Messeri D, Vivarelli A. Priority strategies for sustainable fight against HIV/AIDS in low-income countries. Curr HIV Res. 2004; 2(4):377–93. PMID: 15544458.

4. Kerouedan D. [1986–2006: 20 years of failed international policy to control AIDS in Africa]. Med Trop (Mars). 2007; 67(5):515–28. PMID: 18225738.
5. Assefa Y, Alebachew A, Lera M, Lynen L, Wouters E, Van Damme W. Scaling up antiretroviral treatment and improving patient retention in care: lessons from Ethiopia, 2005–2013. Globalization and Health. 2014; 10:43-. doi: 10.1186/1744-8603-10-43. PMC4046386. PMID: 24886686
6. Kerouedan D. [The Global Fund to fight HIV/AIDS, TB and Malaria 5-y: evaluation policy issues]. Bull Soc Pathol Exot. 2010; 103(2):119–22. doi: 10.1007/s13149-010-0051-2 PMID: 20376595.
7. Piot P, Coll Seck AM. International response to the HIV/AIDS epidemic: planning for success. Bulletin of the World Health Organization. 2001; 79(12):1106–12. PMC2566707. PMID: 11799442
8. WHO. Consolidated guidelines on the use of antiretroviral drugs for treating and preventing HIV infection. Recommendations for a public health approach. Geneva: WHO; 2013.
9. Sheet Fact 2014. UNAIDS; 2014. Global Statistics. Available: http://www.unaids.org/sites/default/files/en/media/unaids/contentassets/documents/factsheet/2014/20140716_FactSheet_en.pdf
10. FHAPCO. Country Progress Report on the HIV Response, 2014. Addis Ababa, Ethiopia: Plan, Monitoring and Evaluation Directorate of FHAPCO, 2014.
11. World Health Organization. HIV/AIDS Factsheet N'3602015 November 2, 2015. Available: http://www.who.int/mediacentre/factsheets/fs360/en/.
12. UNAIDS. Fact sheet—2014 Global Statistics United Nations Joint AIDS Program. 2015 November 12, 2015. Available: http://www.unaids.org/sites/default/files/media_asset/20150714_FS_MDG6_Report_en.pdf.
13. Assefa Y, Alebachew A, Lera M, Lynen L, Wouters E, Van Damme W. Scaling up antiretroviral treatment and improving patient retention in care: lessons from Ethiopia, 2005–2013. Globalization and Health. 2014; 10:43. doi: 10.1186/1744-8603-10-43. PMC4046386. PMID: 24886686
14. FMOH. Health Sector Development Program IV: Annual Performance Report EFY 2006 (2013/14). Addis Ababa, Ethiopia: Federal Ministry of Health (FMOH), 2014.
15. FMOH. HSDP IV Annual Performance Report EFY 2006 Version 1. Addis Ababa, Ethiopia: Federal Democratic Republic of Ethiopia Ministry of Health, 2014.
16. MOH. Health and Health Related Indicators 2003 (2010/11). Addis Ababa, Ethiopia: Federal Ministry of Health, Policy and Planning Directorate, Ethiopia, 2011.
17. Negash T, Ehlers V. Personal Factors Influencing Patients' Adherence to ART in Addis Ababa, Ethiopia. The Journal of the Association of Nurses in AIDS Care: JANAC. 2013. doi: 10.1016/j.jana.2012.11.004 PMID: 23465401.
18. Gusdal AK, Obua C, Andualem T, Wahlstrom R, Tomson G, Peterson S, et al. Voices on adherence to ART in Ethiopia and Uganda: a matter of choice or simply not an option? AIDS care. 2009; 21(11):1381–7. doi: 10.1080/09540120902883119 PMID: 20024714.
19. Markos E, Worku A, Davey G. Adherence to ART in PLWHA at Yirgalem Hosptial, South Ethiopia. Ethiopian Journal of Health Development. 2008; 22(2):174–9.
20. Peltzer K. Patient experiences and health system responsiveness in South Africa. BMC Health Serv Res. 2009; 9:117. doi: 10.1186/1472-6963-9-117 PMID: 19602290; PubMed Central PMCID: PMC2716320.
21. Gilks CF, Crowley S, Ekpini R, Gove S, Perriens J, Souteyrand Y, et al. The WHO public-health approach to antiretroviral treatment against HIV in resource-limited settings. The Lancet. 2006; 368(9534):505–10. doi: 10.1016/S0140-6736(06)69158-7
22. Huerta Munoz U, Källestål C. Geographical accessibility and spatial coverage modeling of the primary health care network in the Western Province of Rwanda. International Journal Of Health Geographics. 2012; 11:40-. doi: 10.1186/1476-072X-11-40 PMID: 22984920.
23. Guagliardo M. Spatial accessibility of primary care: concepts, methods and challenges. International Journal of Health Geographics. 2004; 3(1):3. doi: 10.1186/1476-072X-3-3 PMID: 14987337
24. Luo W, Wang F. Measures of spatial accessibility to healthcare in a GIS environment: Synthesis and a case study in Chicago region. Environment and Planning B. 2003; 30(6):865–84. doi: 10.1068/b29120
25. Gesler W. The uses of spatial analysis in medical geography: a review. Soc Sci Med. 1986; 23(10):963–73. doi: 10.1016/0277-9536(86)90253-4 PMID: 3493536
26. Timyan J, SJ GB, Measham DM, Ogunleye B. Access to Care: More than a Problem of Distance. In: Koblinsky M, Timyan J, Gay J, editors. The Health of Women: A Global Perspective. Colorado: Westview Press; 1993.
27. Vachirasudlekha B. Interdisciplinary HIV care-patient perceptions. International Journal of Health Care Quality Assurance. 2014; 27(5):405. PMID: 25087338

28. Porter MEP. What Is Value in Health Care? The New England Journal of Medicine. 2010; 363 (26):2477–81. 821059531; PMID: 21142528. doi: 10.1056/NEJMp1011024

29. Himakalasa W, Grisurapong S, Phuangsaichai S. Access to antiretroviral therapy among HIV/AIDS patients in Chiang Mai province, Thailand. HIV/AIDS (Auckland, NZ). 2013; 5:205–13. doi: 10.2147/HIV.S49729. PMC3754485.

30. Andaleeb SS. Service quality perceptions and patient satisfaction: a study of hospitals in a developing country. Social Science & Medicine. 2001; 52(9):1359–70. http://dx.doi.org/10.1016/S0277-9536(00)00235-5.

31. Iacobuță A-O. Patients' Evaluation of Access and Quality of Healthcare in EU Countries. A Comparative Analysis. Procedia—Social and Behavioral Sciences. 2012; 62(0):896–900. http://dx.doi.org/10.1016/j.sbspro.2012.09.151.

32. Valentine NB, de Silva A, Kawabata K, Darby C, Murray CJ, Evans DB, et al. Health system responsiveness: concepts, domains and operationalization. Health systems performance assessment: debates, methods and empiricism. 2003:573–96.

33. Donabedian A. An Introduction to Quality Assurance in Health Care. Bashshur R, editor. New York: Oxford University Press; 2003.

34. Valentine N, Darby C, Bonsel GJ. Which aspects of non-clinical quality of care are most important? Results from WHO's general population surveys of "health systems responsiveness" in 41 countries. Social science & medicine. 2008; 66(9):1939–50.

35. Takahashi LM, Rodriguez R. Access Redefined: Service Pathways of Persons Living with HIV and AIDS. Culture, Health & Sexuality. 2002; 4(1):67–83.

36. Penchansky R, Thomas JW. The Concept of Access: Definition and Relationship to Consumer Satisfaction. Medical Care. 1981; 19(2):127–40. PMID: 7206846

37. Tanahashi T. Health Service Coverage and Its Evaluation. Bull World Health Organ. 1978 05/14/2013 2395571]; 56(2):[295–303 pp.]. Available: http://www.ncbi.nlm.nih.gov/pmc/articles/PMC2395571/pdf/bullwho00439-0136.pdf. PMID: 96953

38. Savedoff WD. A Moving Target: Universal Access to Healthcare Services in Latin America and the Caribbean. Washington, DC: Inter-American Development Bank, 2009 January 2009. Report No.: Contract No.: Working Paper #667.

39. McIntyre D, Thiede M, Birch S. Access as a policy-relevant concept in low- and middle-income countries. Health economics, policy, and law. 2009; 4(Pt 2):179–93. doi: 10.1017/S1744133109004836 PMID: 19187569.

40. Gulliford M, Morgan M, Hughes D, Beech R, Figeroa-Munoz J, Gibson B, et al. Access to Health Care London: The Public Health and Health Services Research Group, Department of Public Health Sciences, GKT School of Medicine, King's College London, 2001.

41. Aday LA, Andersen R. A Framework for the Study of Access to Medical Care. Health Services Research. 1974; 9(3):208–20. PMC1071804. PMID: 4436074

42. Gold M. Beyond coverage and supply: measuring access to healthcare in today's market. Health Services Research. 1998; 33(3 Pt 2):625–84. PMC1975648. PMID: 9685110

43. Haddad S, Fournier P. Quality, cost and utilization of health services in developing countries. A longitudinal study in Zaïre. Social Science & Medicine. 1995; 40(6):743–53. http://dx.doi.org/10.1016/0277-9536(94)00134-F.

44. Institute of Medicine Committee on Monitoring Access to Personal Health Care Services. Access to health care in America. Washington, DC: National Academy Press. 1993.

45. CSA. 2007 Population and Housing Census of Southern Nations, Nationalities and Peoples Region of Ethiopia. In: CSA, editor. Addis Ababa, Ethiopia: Central Statistical Agency of Ethiopia; 2009.

46. WODA. Wolaitta 2013. Available: http://www.wolaitta-da.org.et/index.php/wolaitta. Accessed 11 June 2013.

47. Yakob B, Ncama BP. Client satisfaction: correlates and implications for improving HIV/AIDS treatment and care services in southern Ethiopia. Int Health. 2016 April 25, 2016:[1–7 pp.]. Available: http://inthealth.oxfordjournals.org/content/early/2016/03/22/inthealth.ihw008.abstract.

48. Yakob B, Ncama BP. Perceived quality of HIV treatment and care services in Wolaita Zone of southern Ethiopia: a cross-sectional study. BMJ Open. 2015 February 2, 2016; (5:):[e010026 p.]. Available: http://bmjopen.bmj.com/content/5/12/e010026.full.

49. Elashoff JD, Lemeshow S. Sample Size Determination in Epidemiologic Studies. 2005. In: Handbook of Epidemiology. Germany: Springer; [559–94].

50. Valentine N, Tprasas A, Rice N, Narobone S, Chatterji S. Health systems responsiveness: a measure of the acceptability of health-care processes and systems from the user's perspective. In: Smith PC,

Mossialos E, Papanicolas I, Leatherman S, editors. Performance Measurement for Health System Improvement: Expereinces, CHallenges and Prospectus. Cambridge: Cambridge University Press; 2009. p. 138–86.

51. Robone S, Rice N, Smith PC. Health Systems' Responsiveness and Its Characteristics: A Cross-Country Comparative Analysis. Health Services Research. 2011; 46(6 Pt 2):2079–100. doi: 10.1111/j.1475-6773.2011.01291.x. PMC3393001.
52. WHO. Key Informant Opinion Survey [sample report]: World Health Organization Responsiveness Results. World Health Organization. 2003:1–8.
53. Babakus E, Mangold WG. Adapting the SERVQUAL scale to hospital services: an empirical investigation. Health Serv Res. 1992; 26(6):767–86. PMID: 1737708; PubMed Central PMCID: PMC1069855.
54. Parasuraman A, Zeithaml V, Berry L. Refinement and reassessment of the SERVQUAL scale. Journal of retailing. 2004; 67(4):114.
55. Lei P, Jolibert A. A three-model comparison of the relationship between quality, satisfaction and loyalty: an empirical study of the Chinese healthcare system. BMC Health Serv Res. 2012; 12:436. doi: 10.1186/1472-6963-12-436. PMID: 23198824; PubMed Central PMCID: PMC3520735.
56. SDT. The Health Care Climate Questionnaire (HCCQ). PAS–Health Care Climate. July 11, 2014. Available: http://www.selfdeterminationtheory.org/pas-health-care-climate/.
57. The Whoqol G. The World Health Organization quality of life assessment (WHOQOL): Development and general psychometric properties1. Social Science & Medicine. 1998; 46(12):1569–85. http://dx.doi.org/10.1016/S0277-9536(98)00009-4.
58. Liu X, Koirala H. Ordinal Regression Analysis: Using Generalized Ordinal Logistic Regression Models to Estimate Educational Data. Journal of Modern Applied Statistical Methods. 2012; 11(11): 242–54.
59. Winship C, Mare RD. Regression Models with Ordinal Variables. American Sociological Review. 1984 May 20, 2015; 49(August):[512–25 pp.]. Available: http://scholar.harvard.edu/files/cwinship/files/asr_1984.pdf.
60. Hu R, Dong S, Zhao Y, Hu H, Li Z. Assessing potential spatial accessibility of health services in rural China: a case study of Donghai county. International journal for equity in health. 2013; 12:35. doi: 10.1186/1475-9276-12-35 PMID: 23688278.
61. McGrail MR, Humphreys JS. Measuring spatial accessibility to primary care in rural areas: Improving the effectiveness of the two-step floating catchment area method. Applied Geography. 2009; 29 (4):533–41. http://dx.doi.org/10.1016/j.apgeog.2008.12.003.
62. Luo W. Using a GIS-based floating catchment method to assess areas with shortage of physicians. Health and Place. 2004; 10(1):1–11. doi: 10.1016/S1353-8292(02)00067-9 PMID: 14637284
63. Goddard M, Smith P. Equity of Access to Health Care Services: Theory and Evidence from the UK. Social Science & Medicine. 2001 03/23/2013; 53 [1149–62 pp.]. Available: http://www.deepdyve.com/lp/elsevier/equity-of-access-to-health-care-services-75uW81ZZ5a?key=elsevier.
64. Haddad S, Fournier P, Machouf N, Yatara F. What does quality mean to lay people? Community perceptions of primary health care services in Guinea. Social Science & Medicine. 1998; 47(3):381–94. http://dx.doi.org/10.1016/S0277-9536(98)00075-6.
65. Levesque J-F, Harris MF, Russell G. Patient-centred access to health care: conceptualising access at the interface of health systems and populations. International journal for equity in health. 2013; 12:18-. doi: 10.1186/1475-9276-12-18. PMC3610159. PMID: 23496984
66. Yakob B, Ncama BP. Client satisfaction: correlates and implications for improving HIV/AIDS treatment and care services in southern Ethiopia. Int Health. 2016 April 25, 2016:[1–7 pp.]. Available: http://inthealth.oxfordjournals.org/content/early/2016/03/22/inthealth.ihw008.abstract.
67. Tiyou A, Belachew T, Alemseged F, Biadgilign S. Predictors of adherence to antiretroviral therapy among people living with HIV/AIDS in resourcelimited setting of southwest ethiopia. AIDS Research & Therapy. 2010; 7:39–48. doi: 10.1186/1742-6405-7-39 PMID: 55537688.
68. Cook JA, Cohen MH, Burke J, Grey D, Anastos K, Kirstein L, et al. Effects of depressive symptoms and mental health quality of life on use of highly active antiretroviral therapy among HIV-seropositive women. JAIDS Journal of Acquired Immune Deficiency Syndromes. 2002; 30(4):401–9. PMID: 12138346
69. Assefa Y, Lynen L, Wouters E, Rasschaert F, Peeters K, Van Damme W. How to improve patient retention in an antiretroviral treatment program in Ethiopia: a mixed-methods study. BMC Health Services Research. 2014; 14:45-. doi: 10.1186/1472-6963-14-45. PMC3915035. PMID: 24475889
70. Ngowi BJ, Mfinanga SG, Bruun JN, Morkve O. Pulmonary tuberculosis among people living with HIV/AIDS attending care and treatment in rural northern Tanzania. BMC Public Health. 2008; 8:341–7. doi: 10.1186/1471-2458-8-341

71. Clarke TR, Gibson RC, Barrow G, Abel WD, Barton EN. Depression among persons attending a HIV/AIDS outpatient clinic in Kingston, Jamaica. The West Indian Medical Journal. 2010; 59(4):369–73. PMID: 21355510.

72. Masur H, Ognibene FP, Yarchoan R, Shelhamer JH, Baird BF, Travis W, et al. CD4 Counts as Predictors of Opportunistic Pneumonias in Human Immunodeficiency Virus (HIV) Infection. Annals of Internal Medicine. 1989; 111(3):223.

73. Jerene D, Endale A, Hailu Y, Lindtjorn B. Predictors of early death in a cohort of Ethiopian patients treated with HAART. BMC Infect Dis. 2006; 6:136. doi: 10.1186/1471-2334-6-136 PMID: 16948852; PubMed Central PMCID: PMC1569839.

74. Heckman TG. The chronic illness quality of life (CIQOL) model: explaining life satisfaction in people living with HIV disease. Health psychology. 2003; 22(2):140. PMID: 12683734

75. Yakob B, Ncama BP. Perceived quality of HIV treatment and care services in Wolaita Zone of southern Ethiopia: a cross-sectional study. BMJ Open. 2015 February 2, 2016; (5):[e010026 p.]. Available: http://bmjopen.bmj.com/content/5/12/e010026.full.

76. Meessen B, Van Damme W, Tashobya CK, Tibouti A. Poverty and user fees for public health care in low-income countries: lessons from Uganda and Cambodia. The Lancet. 2006; 368(9554):2253–7. http://dx.doi.org/10.1016/S0140-6736(06)69899-1.

77. Dao HT, Waters H, Le QV. User fees and health service utilization in Vietnam: How to protect the poor? Public Health. 2008; 122(10):1068–78. doi: 10.1016/j.puhe.2008.01.002 PMID: 18313088

78. Chaudhuri A, Roy K. Changes in out-of-pocket payments for healthcare in Vietnam and its impact on equity in payments, 1992–2002. Health Policy. 2008; 88(1):38–48. doi: 10.1016/j.healthpol.2008.02.014 PMID: 18423775

79. Souteyrand YP. Free care at the point of service delivery: a key component for reaching universal access to HIV/AIDS treatment in developing countries. AIDS. 2008; 22:161.

80. Yakob B, Ncama BP. A socio-ecological perspective of access to and acceptability of HIV/AIDS treatment and care services: a qualitative case study research. BMC Public Health. 2016; 16(155). doi: 10.1186/s12889-016-2830-6

81. Haggerty JL, Reid RJ, Freeman GK, Starfield BH, Adair CE, McKendry R. Continuity of care: a multidisciplinary review. BMJ: British Medical Journal. 2003; 327(7425):1219–21. PMC274066. PMID: 14630762

82. Wouters E, Heunis C, van Rensburg D, Meulemans H. Patient satisfaction with antiretroviral services at primary health-care facilities in the Free State, South Africa—a two-year study using four waves of cross-sectional data. BMC Health Serv Res. 2008; 8:210. Epub 2008/10/11. doi: 10.1186/1472-6963-8-210 PMID: 18844998; PubMed Central PMCID: PMCPMC2575208.

83. Hekkert KD, Cihangir S, Kleefstra SM, van den Berg B, Kool RB. Patient satisfaction revisited: A multilevel approach. Social Science & Medicine. 2009; 69(1):68–75. http://dx.doi.org/10.1016/j.socscimed.2009.04.016.

84. Osungbade KO, Shaahu VN, Owoaje EE, Adedokun BO. Patients' Satisfaction with Quality of Anti-Retroviral Services in Central Nigeria: Implications for Strengthening Private Health Services. World Journal of Preventive Medicine. 2013; 1(3):11–8.

85. Peltzer K. Patient experiences and health system responsiveness in South Africa. BMC health services research. 2009; 9(1):117.

86. Coyne JS, Hilsenrath P. The World Health Report 2000: Can health care systems be compared using a single measure of performance? American Journal of Public Health. 2002; 92(1):30–3. PMID: 11772753

87. Williams B, Coyle J, Healy D. The meaning of patient satisfaction: An explanation of high reported levels. Social Science & Medicine. 1998; 47(9):1351–9. http://dx.doi.org/10.1016/S0277-9536(98)00213-5.
